# Red Blood Cell Transfusion after Postpartum Hemorrhage: Clinical Variables Associated with Lack of Postpartum Hemorrhage Etiology Identification

**DOI:** 10.3390/jcm12196175

**Published:** 2023-09-24

**Authors:** Francisco Javier Ruiz-Labarta, Rocío Aracil Rodríguez, Ainoa Sáez Prat, Laura Pérez Burrel, Juan Manuel Pina Moreno, Mercedes Sánchez Rodríguez, María Pilar Pintado Recarte, Natalio García-Honduvilla, Miguel A. Ortega, Javier Anguita Velasco, Ana Pérez Corral, Coral Bravo, Juan A. De León-Luis

**Affiliations:** 1 Department of Public and Maternal and Child Health, Faculty of Medicine and Health Science, School of Medicine, Complutense University of Madrid, 28040 Madrid, Spain; franciscojavier.ruiz@salud.madrid.org (F.J.R.-L.); rocioaracilrodriguez@gmail.com (R.A.R.); aino.saez@salud.madrid.org (A.S.P.); lpburrel@salud.madrid.org (L.P.B.); juanmanuel.pina@salud.madrid.org (J.M.P.M.); mariamercedes.sanchez.rodriguez@salud.madrid.org (M.S.R.); ppintadorec@yahoo.es (M.P.P.R.); cbravoarribas@gmail.com (C.B.); jaleon@ucm.es (J.A.D.L.-L.); 2 Department of Obstetrics and Gynecology, University Hospital Gregorio Marañón, 28009 Madrid, Spain; 3 Health Research Institute Gregorio Marañón, 28009 Madrid, Spain; javier.anguita@salud.madrid.org (J.A.V.); apcorral@salud.madrid.org (A.P.C.); 4 Maternal and Infant Research Investigation Unit, Alonso Family Foundation (UDIMIFFA), 28009 Madrid, Spain; 5 Department of Medicine and Medical Specialties, Faculty of Medicine and Health Sciences, University of Alcalá, Alcalá de Henares, 28801 Madrid, Spain; natalio.garcia@uah.es; 6 Ramón y Cajal Institute of Healthcare Research (IRYCIS), 28034 Madrid, Spain; 7 University Center for the Defense of Madrid (CUD-ACD), 28047 Madrid, Spain; 8 Department of Haematology, Hospital General Universitario Gregorio Marañón, Universidad Complutense de Madrid, 28040 Madrid, Spain

**Keywords:** postpartum hemorrhage, severe anemia, maternal morbidity, maternal mortality, red blood cell transfusion

## Abstract

Postpartum hemorrhage (PPH) remains a significant obstetric emergency worldwide and a leading cause of maternal death. However, it is commonly underreported, which can represent a major concern for maternal morbidity and mortality. This retrospective case series study analyzed patients with red blood cell transfusion (RBCt) in the postpartum period over a four-year interval at a specific center. A total of 18,674 patients delivered between January 2018 and December 2021. Patients with postpartum RBCt were classified into two groups: those with identified PPH (i-PPH) and those without (non-i-PPH). Clinical variables, delivery details, blood loss data, and treatment information were collected. Statistical analysis involved a comparison of variables between the i-PPH and non-i-PPH groups. Univariate and multivariate analyses were performed, aiming to identify significant associations between the clinical variables and a lack of PPH identification. The incidence of RBCt was 1.26% (236 cases). Patients receiving RBCt had higher rates of cesarean delivery, twin pregnancy, labor induction, and previous cesarean section. Among patients with postpartum RBCt, 34.3% lacked an identified PPH. The rarity of postpartum RBCt contrasts with the increasing rates of PPH, highlighting the importance of diagnosing PPH and postpartum anemia. A strategy of systematic quantification of blood loss during delivery could help detect PPH and anemia before adverse consequences occur.

## 1. Introduction

The need for transfusion of blood components in the treatment of postpartum hemorrhage (PPH) is a rare event (0.9 to 2.5% of all deliveries) [1], despite the current increase in the incidence of PPH worldwide [2]. The vast majority of cases of red blood cell transfusion (RBCt) in the postpartum period occur as a consequence of acute and severe anemia secondary to PPH. It is important to diagnose postpartum anemia because it is associated with important maternal morbidities, including depression, fatigue, and impaired cognition [3]. Additionally, these adverse events can negatively impact mother–child bonding and the mother’s ability to care for the newborn child. Even severe maternal morbidity, such as myocardial ischemia, has been described as a frequent complication of PPH, and suboptimal transfusion therapy may lead to maternal death [4]. Therefore, it is important to identify patients who need transfusion support and to ensure this is adequate.

PPH remains a common obstetric emergency and is the leading cause of maternal mortality worldwide [5]. PPH-related deaths are potentially preventable with timely diagnosis and management [6]. There is currently no single satisfactory definition of PPH. PPH is commonly defined as blood loss of 500 mL or more within 24 h after vaginal birth or >1000 mL after cesarean section, while severe PPH is defined as blood loss of 1000 mL or more and massive life-threatening PPH as ongoing blood loss of more than 2500 mL or hypovolemic shock within the same timeframe [6]. Other definitions include a decrease in hematocrit by more than 10% compared to the baseline value, or a decrease in hemoglobin value by 4 g/dL compared to pre-delivery values [6]. The overall global incidence of PPH is estimated to be 6–11%, and that of severe PPH is 1–3%, with substantial variations across regions; however, the true incidence of PPH is likely to be much higher than reported [7]. Uterine atony is the most common cause of PPH, and other causes include genital tract trauma (i.e., vaginal or cervical lacerations), uterine rupture, retained placental tissue, and maternal coagulation disorders [8].

Clinical events with RBCt require special attention from professionals because they can be accompanied by severe maternal morbidity [9,10]. However, in some cases, they represent a critical event that can be prevented. Therefore, patient safety experts recommend conducting an audit of clinical records of patients with RBCt to find areas for improvement in their management.

In our center, we observed several cases in which a PPH event was not described in the delivery; however, during subsequent hospitalization, the patient presented with symptoms of dizziness and laboratory-confirmed severe anemia that required RBCt. In medicine, false negatives (not identifying a PPH in our case) pose a problem since they can increase maternal morbidity and mortality. For this reason, this study was designed to audit the clinical history of each patient with postpartum RBCt in our center, analyzing the clinical-analytical variables to find possible points of improvement in maternal-perinatal care. The objectives of this study were (a) to carry out a descriptive analysis of the clinical-analytical variables of the cohort of patients with postpartum RBCt in our center; (b) to analyze how these variables are distributed between two study groups, namely, patients with identification of a PPH (i-PPH) and patients without identification of a PPH (non-i-PPH); and (c) to identify the clinical-analytical variables that allow the prediction of which patients require postpartum RBCt despite a PPH not being identified (non-i-PPH).

## 2. Materials and Methods

This was a retrospective case series study of patients with RBCt in the postpartum period and was carried out by means of a hospital-based cohort composed of all patients who delivered at the Obstetrics and Gynecology Department of the Hospital general universitario Gregorio Marañon between 1 January 2018 and 31 December 2021. The list of cases was extracted from the blood transfusion database of the hospital’s Hematology Service. The Institutional Review Board approved our study protocol (HOS).

Patients with postpartum RBCt were classified into two groups: those with identified PPH of any etiology (i-PPH) and those without a mention of HPP or an etiology for anemia in their clinical record (non-i-PPH). If the identification of a PPH was described, information on the type of etiology was collected, as was information on the treatments administered.

It was verified that the indication for RBCt was determined per the Patient Blood Management recommendations of the main international guidelines, mainly hemoglobin < 7 g/dL with or without clinical symptoms [3], and the assessment of PPH was performed according to our institutional obstetrics hemorrhage protocol approved in 2016. According to this protocol, which is based on international guidelines [8], when a patient begins postpartum bleeding, the obstetrician performs a systematic clinical examination aiming at etiological diagnosis, with a review of the integrity of the placenta and membranes (including an ultrasound study if necessary), the integrity of the soft birth canal, the status of uterine contraction, and bladder catheterization. The causes of PPH (uterine atony, birth canal trauma, tissue retention, or coagulation alteration) are clearly established in our protocol in accordance with the main international guidelines [8], as well as the standardization of appropriate treatment. In our center, during the study period, the quantification of postpartum blood loss was not carried out systematically in all deliveries. Our protocol establishes the need to examine patients’ clinical condition (using patients’ vital signs and symptoms) to assess the severity of PPH in relation to the degree of shock. Patients are managed by a multidisciplinary team comprising obstetrics and anesthesiology staff [11].

First, we collected data on the following clinical variables from all patients in the hospital cohort who delivered at our center during the study period: maternal age (years), type of gestation (singleton or multiple), patients with previous C-section, gestational age at delivery (weeks), labor induction, type of delivery (vaginal or C-section), and newborn weight (grams).

Then, we collected data on the following clinical variables from the series of patients with postpartum RBC: maternal age (years), country of origin, parity, patients with previous C-section, patients with multiple gestation, and patients with HTN/preeclampsia. Information on the following variables was collected: data in relation to delivery and the newborn (gestational age (weeks), labor induction, C-section, rate of live NB, weight of the NB, pH of the NB and Apgar test), variables in relation to the loss of hematic and iron therapy (predelivery hemoglobin/hematocrit/platelets/INR, pretransfusion hemoglobin/platelets, posttransfusion hemoglobin/platelets, number of red blood cell (RBC) units transfused, IV iron administration), stay at the Postanesthesia Resuscitation Unit (PARU), and hospital stay. Finally, information on complications related to the transfusion was sought. Within the subgroup of patients with vaginal delivery, the rates of eutocic and instrumental delivery, episiotomy, perineal tears, and tears without episiotomy were determined.

All data pertaining to the study variables were documented in an Excel (Microsoft Office LTSC Professional Plus 2021) sheet. Quantitative variables are expressed as mean ± standard deviation (SD); qualitative variables are expressed as numbers (percentages). Statistical analysis was performed using the SPSS software package, version 25 (IBM Co., Somers, NY, USA), with its default settings. The distribution of the variables between the 2 study groups (i-PPH and non-i-PPH) was analyzed. Student’s *t*-test was used to compare the median values of the quantitative variables, and Fisher’s exact test or chi-squared test was used to compare the qualitative variables. Finally, variables with clinical relevance and/or a *p*-value equal to or less than 0.20 in the univariate analysis were included in the multivariate logistic regression analysis to determine the adjusted effect of these variables in their relationship with the absence of identification of a PPH (non i-PPH). Odds ratios (ORs) with 95% confidence intervals (CIs) were calculated. *p* < 0.05 was used to indicate a statistically significant difference.

## 3. Results

A total of 18,674 patients delivered at our center during the 4-year study period. In this patient population, the mean maternal age was 33.1 years. There were 4.9% of patients with multiple gestation, and 10% of patients had a previous C-section. The mean gestational age at delivery was 38.6 weeks, 20% of patients had labor induction, and 20% of patients delivered by C-section, with a mean weight of 3220 g among the newborns.

In this sample of patients, there were 236 (1.26% of all deliveries) patients with postpartum RBCt, whose data were extracted. They were classified into two study groups: 155 (65.7%) patients in whom a PPH was identified (i-PPH) and 81 patients (34.3%) in whom a PPH was not identified (non-i-PPH).

Table 1 describes the distribution of the maternal clinical variables, the characteristics of childbirth, and those related to blood loss among the patients with postpartum RBCt, as well as the distribution between the study groups (i-PPH and non-i-PPH).

No side effects were described in relation to RBCt, and there was only one case of maternal death due to a massive retroperitoneal hematoma and disseminated intravascular coagulation after urgent cesarean section, in which 37 packed red blood cells were transfused.

In the comparison of the series of patients with postpartum RBCt with the hospital-based cohort, we found statistically significant differences (*p* < 0.05) for the C-section rate (36% vs. 20%), twin pregnancy rate (10.2% vs. 4.9%), labor induction rate (37.7%; vs. 20%), and percentage of patients with previous C-section (25% vs. 10%).

Within the series of patients with postpartum RBCt, the clinical-analytical variables that presented statistically significant differences (*p* < 0.05) between the study groups (i-PPH vs. non-i-PPH) were maternal age (33.6 vs. 31.4 years), C-section (40.6% vs. 27.2%), mean prepartum hemoglobin (11.1 vs. 10.4 g/dL), mean pretransfusion hemoglobin (7.5 vs. 7.1 g/dL), mean net blood cell (RBC) units transfused per patient (3.2 vs. 2.1), stay at the PARU (71.6% vs. 22.2%), and mean hospital stay (5.3 vs. 3.8 days).

Since some variables, such as the rates of episiotomies and tears, were the main variables related to vaginal deliveries, cesarean sections were excluded from the univariable analysis of the subgroup of patients with vaginal delivery, which is presented in Table 2. There was a total of 151 patients with postpartum vaginal RBCt. The variables with statistically significant differences (*p* < 0.05) between the study groups (i-PPH vs. non-i-PPH) were eutocic delivery (62% vs. 40.7%), instrumental delivery (38% vs. 59.3%), and episiotomy (58.7% vs. 74.6%). Up to 14.6% of vaginal deliveries were torn without episiotomy, but we did not find significant differences between the groups.

**Figure 1:**
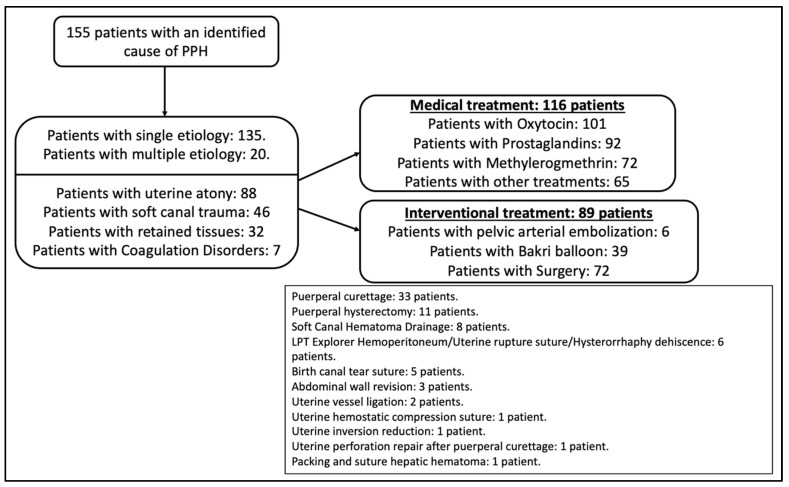
Treatments administered to transfused patients in the postpartum period in whom a cause of PPH was identified.

Within the group of patients with postpartum RBCt in whom PPH was identified (N = 155), the most frequent cause identified was atony (50.9%), followed by trauma (26.6%), retained tissues (18.5%), and coagulation disorders (4%). The most widely used uterotonic medical treatment was oxytocin, followed by prostaglandins. Within the interventional treatment, there were 6 cases of uterine artery embolization, 39 cases of Bakri balloon placement, and 72 cases of surgery, with puerperal curettage being the most performed procedure (45.8%), followed by puerperal hysterectomy (15.3%). Figure 1 shows all the treatments administered to these patients.

Finally, Table 3, which shows the multivariate logistic regression analysis carried out with the total number of patients in the study and the evaluation of the differences between the study groups (i-PPH and non-i-PPH), shows that parity was significantly associated with the absence of described PPH; in contrast, antepartum Hb and C-section were significantly associated with the identification of PPH. Likewise, in the subanalysis of the group of patients with vaginal delivery, parity and instrumental delivery were significantly associated with the absence of PPH identification; in contrast, antepartum Hb was significantly associated with the identification of HPP.

## 4. Discussion

The results obtained in our center after attending the deliveries of 18,674 patients over a period of 4 years show that postpartum RBCt is a rare event (1.26%; 95% CI (1.10–1.42)). With postpartum RBCt, the rates of C-section, twin pregnancy, labor induction, and patients with previous C-section were significantly higher than those of the total population. Parity and instrumental delivery being the variables significantly associated with the absence of PPH identification, whereas C-section and prepartum hemoglobin were significantly associated with the identification of PPH.

According to the data analyzed, in our center, one patient was transfused for every 79 deliveries. This transfusion rate is similar to that of other studies published in the US population [12] and is higher than that published by studies in the European population [13] (although these studies show great variability with rates ranging from 1 in 90 deliveries to 1 in 476 deliveries depending on the country). In recent years, a clear increase in the rate of transfusions in the perinatal period has been published, which seems to be justified by the current increase in the rate of C-sections, twin gestations, labor inductions, and placenta accreta spectrum [12]. We understand that the transfusion rate in our hospital is high, and this could be because we are a reference center for pregnancies with serious maternal and fetal pathologies that represent a higher percentage of patients with placentation disorders, previous C-section, twin pregnancies, and an increased labor induction rate.

In the series of patients with postpartum RBCt, a significantly higher rate was found, with respect to the hospital cohort, regarding variables that are risk factors for PPH (C-section, twin pregnancy, labor induction, and patients with previous C-section), which is in accordance with what has been published in the literature [11,14]. This highlights the importance of correctly assessing patients with risk factors for postpartum hemorrhage in terms of proper management of anemia prior to delivery, as well as carrying out early identification and treatment of hemorrhage, which reduces the need for blood transfusion.

As we highlighted in the introduction, the audit of cases in which RBCt is performed can help to find points of improvement as strategies to prevent further morbidities. At this point, we are struck by the fact that among the 34.3% of patients with postpartum RBCt, a PPH event was not identified in the medical records of those with a severe anemia subsidiary that would justify blood transfusion. In addition, we were able to find significant differences between the study groups (i-PPH and non-i-PPH) showing a higher mean number of red blood cell (RBC) units transfused per patient, a higher percentage of patients with a stay at the PARU, and a longer average length of hospital stay among the patients with an identification of PPH (i-PPH). This seems to indicate that PPH is identified in the most serious cases, and consequently, patients need a longer stay in units with exhaustive monitoring (e.g., the PARU), more blood transfusions, and more days of recovery until discharge.

The multivariate analysis revealed that the variables associated with the lack of identification of a PPH were instrumental delivery (OR 2.75. CI 1.21–6.27) and parity (OR 2.72. CI 1.33–5.55). Instrumental delivery occurred in 46.4% of the patients with vaginal delivery and RBCt, with an episiotomy in 94.2% of these patients and forceps being the instrument most often used (Table 2). In our center, forceps have traditionally been the most widely used surgical instrument, and the professionals at our center have extensive experience with their use [15]. 

Instrumental deliveries increase the probability of a perineal or vaginal tear. Blood loss associated with these lesions may be underestimated if insufficient attention is paid to the amount of bleeding from the birth canal while revision and repair are performed.

Despite the fact that parity appears in the literature as a risk factor for PPH due to uterine atony [16], in our study, it is associated with the lack of identification of a PPH, and we do not know when this has special relevance. If it is during the nonimmediate postpartum period, a possible explanation is that multiparous women present more abundant hematic lochia during the hours following delivery, and this blood loss may go unnoticed. This reinforces the idea highlighted in the recommendations of international guidelines that it is necessary to pay attention to the quantification of blood loss, both at the time of delivery and in the hours after it, during admission to puerperium [17].

In contrast, variables that were associated with the identification of a PPH in the multivariate analysis were cesarean delivery (OR 0.42; CI 0.22–0.81) and prepartum hemoglobin (OR 0.66; CI 0.53–0.82). Cesarean delivery occurred in 36% of all patients with postpartum RBCt, with 40.6% being in the i-PPH group and 27.2% in the non-i-PPH group (*p* < 0.05) (Table 1). This may be because C-section is associated with causes of bleeding (e.g., placentation disorders, atony, uterine rupture, and coagulation disorders) and occurs in a more controlled and monitored environment, with greater vigilance on the part of the obstetrics, anesthesia, and nursing team, which allows better identification of blood loss and, therefore, the cause of PPH. 

Low prepartum hemoglobin levels seem to be associated with the absence of HPP identification in the study group. These patients may need a postpartum RBCt even without striking blood loss. This situation highlights the importance of proper management of anemia during pregnancy so that patients reach childbirth with optimal hemoglobin levels, which minimizes the chance of a transfusion [3].

We wish to emphasize that, based on all of the above findings, we are concerned that a PPH was not identified in one third of patients with postpartum RBCt since this implies a notable false-negative rate for the identification of postpartum bleeding in our usual clinical practice, which can be associated with patient morbidity and mortality. Therefore, it is essential to identify this group of patients and generate different clinical tools that help us improve their diagnosis. At this point, we must say that in our center, a quantification of blood loss in deliveries was not carried out systematically during the study period, which could lead to the lack of identification of blood loss and, consequently, inability to identify a certain etiology. Because of this, since the end of 2021 in our center, we have implemented a protocol for gravimetric quantification of blood loss to be applied systematically in all deliveries [18]. This will allow us to analyze in the future whether this clinical tool will help improve the identification of PPH and its etiology, provide early treatment and reduce morbidity and mortality in patients, an aspect that has not been demonstrated at the moment according to the studies conducted by the ACOG Committee [17].

There are studies published in the literature that report the clinical experience of multiple centers regarding blood transfusion in patients with postpartum hemorrhage [19,20,21]. However, we did not find any study like ours that compares the clinical results of patients with postpartum RBCt depending on whether PPH has been identified. 

Among the limitations of this study are its retrospective nature and the fact that a comparison with similar studies assessing the risk factors for unrecognized HPP in patients with postpartum RBCt is not possible. More studies should be carried out in this group of patients to elucidate preventable strategies.

Some of the strengths of this study reside in the fact that it was performed in a hospital-based population with more than 200 cases of postpartum RBCt. A register of the HPP event and its management was recorded attending to the possible etiologies. After this study, we are able to identify which factors may be associated with an unrecognized HPP that requires transfusion and suggest strategies for its prevention such as blood loss quantification, which is now carried out in our hospital. 

## 5. Conclusions

In our center, postpartum RBCt is a rare event (1.26%) that occurs more frequently in patients with risk factors for PPH (C-section, twin pregnancy, labor induction, and previous cesarean section). In 34.3% of the patients with postpartum RBCt, no PPH is identified in their clinical record. Higher parity and instrumental delivery are independently and significantly associated with this fact. As a lack of early PPH identification may lead to adverse maternal outcomes, attention should be paid to multiparous patients and those with instrumental delivery to prevent an unrecognized hemorrhage and subsequent anemia. We suggest blood loss quantification during childbirth as a strategy for early diagnosis and intervention. Every center should audit their protocols for PPH detection and treatment, including their transfusion policy, in order to identify their own improvement areas.

## Figures and Tables

**Table 1:** Distribution of maternal clinical variables, delivery characteristics, and those related to blood loss, in relation to the total number of pregnant women transfused and between the study groups. HTC, hematocrit; Hb, hemoglobin; HTN, hypertension; PACU, post-anesthesia care; RBC, red blood cells.

Variable	Total (N = 236)	Non-Identified HPP. Non-i-PPH (N = 81)	Identified HPP. i-PPH (N = 155)	*p*-Value
Maternal and gestational clinical variables				
Maternal age (years)	32.9 ± 6.2	31.4 ± 6.8	33.6 ± 5.8	0.009
Primiparity	160 (67.8%)	60 (74.1%)	100 (64.5%)	0.135
Patients with previous C-section	59 (25%)	16 (19.8%)	43 (27.7%)	0.178
Patients with multiple gestation	24 (10.2%)	5 (6.2%)	19 (12.3%)	0.176
Patients with HTN/preeclampsia	38 (16.1%)	8 (9.9%)	30 (19.3%)	0.064
Characteristics of childbirth and newborn				
Gestational age (weeks)	37.6 ± 3.4	37.6 ± 3.7	37.7 ± 3.3	0.83
Labor induction	89 (37.7%)	29 (35.8%)	60 (38.7%)	0.674
C-Section	85 (36%)	22 (27.2%)	63 (40.6%)	0.04
Living newborn	229 (97%)	80 (98.8%)	149 (96.1%)	0.427
Weight (grams)	3064.5 ± 760.1	3054.8 ± 811.6	3069.5 ± 734.5	0.890
pH	7.24 ± 0.1	7.24 ± 0.1	7.23 ± 0.1	0.462
Apgar 0	8.2 ± 1.7	8.2 ± 1.8	8.1 ± 1.7	0.869
Apgar 5	9.4 ± 1.2	9.4 ± 1.1	9.4 ± 1.2	0.667
Blood loss, iron therapy, and hospital stay				
Hb prepartum	10.9 ± 1.6	10.4 ± 1.5	11.1 ± 1.6	0.001
Anemia (Hb < 11 g/dL)	110 (46.6%)	50 (61.7%)	60 (38.7%)	0.001
Pre-delivery HTC	33.1 ± 4.4	31.3 ± 3.7	34.1 ± 4.5	<0.001
Pre-delivery platelets	225.2 ± 91.7	255.5 ± 97.3	206.8 ± 83.3	<0.001
Pre-delivery INR	1 ± 0.1	0.98 ± 0.1	1 ± 0.2	0.440
Pre-transfusion Hb	7.3 ± 1.2	7.1 ± 0.8	7.5 ± 1.3	0.034
Pre-transfusion platelets	200 ± 95.5	237.2 ± 111.2	179.3 ± 78.7	<0.001
No. RBC concentrates transfused per patient	2.8 ± 3.1	2.1 ± 0.5	3.2 ± 3.8	0.012
RBC concentrate transfusion > 2	57 (24.1%)	10 (12.3%)	47 (30.3%)	0.002
Post-transfusion Hb	9.2 ± 1.1	8.9 ± 0.8	9.3 ± 1.2	0.005
Post-transfusion platelets	197.9 ± 96	230.2 ± 100.6	180.9 ± 89.2	<0.001
Iron IV	58 (24.6%)	15 (18.5%)	43 (27.7%)	0.152
PACU stay	129 (54.7%)	18 (22.2%)	111 (71.6%)	<0.001
Admission days	4.8 ± 4.3	3.8 ± 2.6	5.3 ± 4.9	0.010
Stay ≥7 days	42 (17.8%)	7 (8.6%)	35 (22.6%)	0.007

**Table 2:** Distribution of the study variables in relation to vaginal deliveries in the total number of pregnant women transfused and between the study groups.

Variable	Total (N = 151)	Non-Identified HPP Non-i-PPH (N = 59)	Identified HPP. i-PPH (N = 92)	*p*-Value
Eutocic delivery	81 (53.6%)	24 (40.7%)	57 (62%)	0.01
Instrumental Childbirth	70 (46.4%)	35 (59.3%)	35 (38%)	0.01
Forceps	62 (41.1%)	34 (57.6%)	28 (30.4%)	
Sucker	4 (2.6%)	1 (1.7%)	3 (3.3%)	
Spatulas	4 (2.6%)	0 (0%)	4 (4.3%)	
Episiotomy	98 (64.9%)	44 (74.6%)	54 (58.7%)	0.04
Tear	53 (35.1%)	25 (42.4%)	28 (30.4%)	0.13
Tear without episiotomy	22 (14.6%)	7 (11.9%)	15 (16.3%)	0.45

**Table 3:** Back-step multivariate logistic regression analysis of factors associated with the lack of identification of PPH in transfused patients during the postpartum period.

Variables	OR (95% Confidence Interval)	*p*-Value
Patients with RBCt (N = 236)		
Parity	2.72 (1.33–5.55)	0.006
Prepartum Hb	0.66 (0.53–0.82)	0.001
C-section	0.42 (0.22–0.81)	0.009
Patients with vaginal delivery (N = 151)		
Parity	4.23 (1.67–10.72)	0.002
Prepartum Hb	0.61 (0.45–0.81)	0.001
Instrumental delivery	2.75 (1.21–6.27)	0.016

## Data Availability

The datasets used and/or analyzed during the present study are available from the corresponding author upon reasonable request.
